# Discogenic Low Back Pain: Anatomic and Pathophysiologic Characterization, Clinical Evaluation, Biomarkers, AI, and Treatment Options

**DOI:** 10.3390/jcm13195915

**Published:** 2024-10-03

**Authors:** Matteo De Simone, Anis Choucha, Elena Ciaglia, Valeria Conti, Giuseppina Pecoraro, Alessandro Santurro, Annibale Alessandro Puca, Marco Cascella, Giorgio Iaconetta

**Affiliations:** 1 Department of Medicine, Surgery and Dentistry “Scuola Medica Salernitana”, University of Salerno, Via S. Allende, 84081 Baronissi, Italy; eciaglia@unisa.it (E.C.); vconti@unisa.it (V.C.); asanturro@unisa.it (A.S.); apuca@unisa.it (A.A.P.); giaconetta@unisa.it (G.I.); 2 BrainLab S.R.L., Mercato San Severino, 84085 Salerno, Italy; pecoraropina@gmail.com; 3 Neurosurgery Unit, University Hospital “San Giovanni di Dio e Ruggi, D’Aragona”, 84131 Salerno, Italy; 4 Department of Neurosurgery, Aix Marseille University, APHM, UH Timone, 13005 Marseille, France; anis.c13@gmail.com; 5 Laboratory of Biomechanics and Application, UMRT24, Gustave Eiffel University, Aix Marseille University, 13005 Marseille, France; 6 Clinical Pharmacology Unit, University Hospital “San Giovanni di Dio e Ruggi, D’Aragona”, 84131 Salerno, Italy; 7 Legal Medicine Unit, University Hospital “San Giovanni di Dio e Ruggi, D’Aragona”, 84131 Salerno, Italy

**Keywords:** LBP, low back pain, pathophysiology, clinical trajectory, biomarkers, treatment, AI, pain matrix, discogenic LBP

## Abstract

Discogenic low back pain (LBP) is a significant clinical condition arising from degeneration of the intervertebral disc, a common yet complex cause of chronic pain, defined by fissuring in the annulus fibrosus resulting in vascularization of growing granulation tissue and growth of nociceptive nerve fibers along the laceration area. This paper delves into the anatomical and pathophysiological underpinnings of discogenic LBP, emphasizing the role of intervertebral disc degeneration in the onset of pain. The pathogenesis is multifactorial, involving processes like mitochondrial dysfunction, accumulation of advanced glycation end products, and pyroptosis, all contributing to disc degeneration and subsequent pain. Despite its prevalence, diagnosing discogenic LBP is challenging due to the overlapping symptoms with other forms of LBP and the absence of definitive diagnostic criteria. Current diagnostic approaches include clinical evaluations, imaging techniques, and the exploration of potential biomarkers. Treatment strategies range from conservative management, such as physical therapy and pharmacological interventions, to more invasive procedures such as spinal injections and surgery. Emerging therapies targeting molecular pathways involved in disc degeneration are under investigation and hold potential for future clinical application. This paper highlights the necessity of a multidisciplinary approach combining clinical, imaging, and molecular data to enhance the accuracy of diagnosis and the effectiveness of treatment for discogenic LBP, ultimately aiming to improve patient outcomes.

## 1. Introduction

Low back pain (LBP) is a frequent, disabling clinical entity covering a spectrum of different types of conditions and manifesting with pain within a topographic region extending from the 12th rib to the iliac crest. Significantly, in 2020, 619 million people worldwide suffered from LBP (almost 10% of the world’s population), and this number is expected to reach 843 million by 2050 [1], featuring “the global epidemic of low back pain” [2]. The same consortium just mentioned regarding the Western European geographical area alone, for example, estimates the rate of years lived with disabilities (YLDs) per 100,000 to be about 1070 (737–1360). Equally high are the data from the high-income areas of the United States, which are worth about 1060 (YLDs per 100.000). Interestingly, these data are much higher than the global data (832 YLDs per 100,000). This evidence offers interesting insights into the preventive strategies that need to be implemented. Thus, it is well understood how the impact of the disease is considerable both in personal terms, on the individual patient, and in terms of the costs to society as well. Consequently, the economic burden of the disease is considerable [3,4].

The term LBP encompasses a set of acute and chronic clinical conditions with complex pathophysiology and multiple etiologies [5,6,7]. This issue often complicates the diagnosis [5]. Causes of LBP include nonspinal or spinal conditions. Nevertheless, non-specific LBP is defined as LBP that cannot be linked to a specific or identifiable pathological or anatomical entity [8]. As a result, only a small percentage (about 20%) of LBP cases can be attributed with reasonable certainty to a pathological or anatomical entity.

Discogenic back pain (DBP) is a complex form of back pain caused by intervertebral disc (IVD) pathology. Although there may be no radiographic signs of disc herniation compressing the spinal column or nerves, DBP is primarily linked to disc degeneration. Nevertheless, within the mechanisms of DBP, other contributing factors include biomechanical instability, localized inflammation, vertebral endplate damage, and the reinnervation of the disc area with pain-sensitive F [9]. Due to this complex pathophysiology, effective clinical management is often challenging [10,11]

This narrative review focuses on LBP as a symptom of structural damage to the intervertebral disc (IVD), providing expert opinions on emerging research trajectories bridging bench research to clinical settings. The IVD, mistakenly thought to be a mere static component that responds passively to load stresses, instead possesses a complex articulated tissue biology with interesting implications for its interaction with other structures of the spine.

## 2. IVD Anatomy and Morphological Alterations

The intervertebral disc (IVD) is a fibrocartilaginous junction that connects two adjacent vertebrae in the spinal column. IVD is placed between two vertebral bodies, contained between the margins of adjacent vertebrae (disc plates). In detail, IVD forms a complex system, and its structure is essentially represented by three distinct components [12]:

- A central nucleus pulposus (NP);
- A peripheral annulus fibrosus (AF);
- Two vertebral end-plates (VEPs).

Specifically, NP represents the core of IVD and consists of a spheroidal gelatinous mass. It is composed of a mucoprotein gelatinous structure containing approximately 66% to 86% water, type-II collagen fibrils, and proteoglycans. Between proteoglycans, aggrecan and versican act by binding to hyaluronan, as well as several small leucine-rich proteoglycans. The proteoglycans, as glycosaminoglycan (GAG) chains of chondroitin sulfate and keratan sulfate, are largely responsible for retaining water within the NP. Additionally, NP contains a low density of small chondrocyte-like cells producing the extracellular matrix (ECM) products and maintaining the integrity of the NP. Thereby, the NP’s function, as an inner gel-like center, is to absorb and uniformly redistribute the load stresses to the periphery, avoiding excessive pressure on the AF [13,14,15]. AF is an outer fibrous ring and consists of 15 to 25 stacked sheets of stratified elastic tissue, organized in concentric rings (laminae or lamellae). It is composed externally of Sharpey’s type I collagen fibers, with outer lamellae continuing into the longitudinal ligaments and vertebral bodies. The AF central part is composed of an extracellular matrix enriched by chondrocytes and a series of protein fibers (mainly type II collagen) arranged in an alternate pattern, therefore not oriented vertically, allowing for greater effective resistance. The lamellae are also interconnected through translamellar bridges, which permit balance between strength and flexibility. In general, AF’s structure contains the NP and prevents the development of stress concentrations which could cause damage to the underlying vertebrae or their end plates [16]. The third element of the IVD, VEP, consists of an upper and a lower cartilaginous end-plate approximately 0.6 mm thick, covering the superior and inferior part of the disc, with calcified cartilage adjoining the bone. The endplate permits diffusion and provides the main source of nutrition for the disc [12]. Indeed, the IVD is innervated only in the outer third of the AF and is largely avascular, with no major arterial branches to the disc, and only the outer AF is vascularized and supplied by small arteries. Blood vessels near the disc–bone junction of the vertebral body, as well as those in the outer annulus, supply the NP and inner AF, especially in young people. Glucose, oxygen, and other nutrients reach the avascular regions by diffusion. The same process removes metabolites [17].

**Figure 1:**
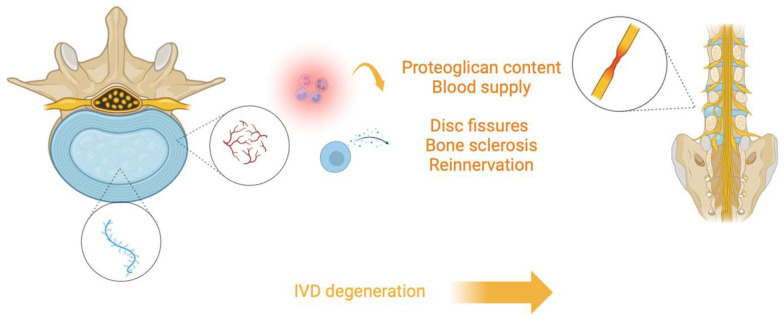
Illustration depicting the process of intervertebral disc (IVD) degeneration. The central nucleus pulposus (blue) loses proteoglycan content and blood supply, leading to structural changes such as disc fissures, bone sclerosis, and reinnervation. These degenerative changes contribute to the breakdown of the spinal structure, ultimately resulting in the degeneration of the IVD.

Given its anatomy, IVD is a highly specialized and organized tissue that is normally well integrated with its adjacent tissues. However, changes in IVD structure can be observed in different conditions. For example, vessels can recede, determining less direct blood supply in the IVD, while changes and diminutions in proteoglycan content can determine water loss from the matrix. Moreover, the cartilage of VEPs can undergo thinning, altered cell density, formation of fissures, and sclerosis of the subchondral bone. Furthermore, NP can appear less gelatinous and more fibrous, with possible cracks and fissures, and AF lamellar organization can undergo remodeling with more bifurcation and interdigitations and increasing lamellae thickness (Figure 1).

These changes are similar in aging and several pathological–degenerative conditions involving IVD, causing discussion as to whether aging and degeneration are separate processes or the same process occurring over a different timescale. Whatever the initial cause, a change in the morphology of the tissue is inevitably related to changes in the physiologic and mechanical functioning of the IVD [18,19,20].

## 3. Pathophysiology

Throughout life, the IVD undergoes biological changes that lead to its degeneration. The NP shifts from a jelly-like structure to fibrous tissue due to proteoglycan loss and extracellular matrix alterations, which decrease water content. This results in a reduction in disc height, flexibility, and hydration. With age, the population of progenitor cells in the NP declines, and senescent cells increase, contributing to inflammation and degeneration. Senescence-associated secretory phenotype (SASP) enhances inflammatory cytokine production, which is linked to discogenic pain [15]. Metabolic shifts and mitochondrial dysfunction further exacerbate degeneration, along with cellular processes like pyroptosis and autophagy. Advanced glycation end products (AGEs) also induce cytotoxicity, linking disc degeneration to conditions like diabetes. Recent research indicates that microRNAs play a role in regulating cellular responses and extracellular matrix metabolism, influencing intervertebral disc degeneration. Thus, targeting senescence, metabolic dysfunction, and molecular regulators like microRNAs may offer therapeutic approaches to address disc degeneration.

### 3.1. Intervertebral Disc Degeneration versus Aging

Preclinical investigations showed that one of the earliest changes in IVD degeneration (IVDD) is a loss of content and composition of major disc proteoglycan, aggrecan, resulting in reduced balance hydration, height, and flexibility of the disc [21]. Furthermore, molecular evidence suggests that the different cell morphologies and immune phenotypes in the NP reflect progressive phases of degeneration during aging. Murine studies have illustrated how the population of NP progenitor cells expressing the G-protein-coupled receptor Uts2R, the receptor tyrosine kinase (Tie2), and disialoganglioside 2 (Gd2) declines with age in the IVD, while the population of chondrocyte-like (CL) cells becomes more prominent over time [22]. Based on these findings, while a correlation between decreased numbers of UTS2R+ or TIE2+/GD2+ stem-like cells and the onset of IVDD can be postulated, this process has not been specifically described for human NP CL cell populations of various degrees of IVDD. Moreover, it is unclear whether these changes are the results of adaptations to the altered environment of the growing spine.

As the stem compartment decreases, an increasing number of senescent cells is observed with advancing age and degeneration of disc tissue. This is obvious, since senescence evolved as an initial protective mechanism to maintain cellular homeostasis when threatened by different types of stress, including mitochondrial dysfunction, telomeric erosion, persistent DNA damage, oxidative stress, and inflammation [23,24]. However, when the senescent pool persists because the regenerative capacity of the tissue is exceeded, the senescence phenotype becomes aberrant through the senescence spreading to neighboring cells and the sustainment of a low-grade inflammatory state through the SASP. Indeed, enhanced expression of pro-inflammatory cytokines (interleukin (IL)-1, MMP10, MMP12, cyclooxygenase 2 (COX2), IL8, IL10, IL2, and IL17) has been strongly associated with discogenic pain [25].

Regardless of the types of damage that have been implicated in driving disc aging, some common molecular and cellular signatures of senescence have been found in IVDD. Expression of the cell cycle arrest protein p16^INK4a^, a well-known senescent marker, increases with age in the discs of patients and is positively correlated with the expression of matrix metalloproteinases, putatively bursting extracellular matrix (ECM) degeneration [26]. The percentage of SA-βGal+ senescent disc cells also correlates with the degeneration degree of disc tissue and, more importantly, negatively correlates with Ki67-positive proliferating cells, clearly suggesting a deep impact on tissue functionality [27]. The primary senescent disc cells also induce immune cell recruitment in a vain attempt to remove the aberrant products. These further fuel the inflammatory microenvironment of the disc. Because of these detrimental changes, the adjunct inability of the mature avascular disc to remove and replace accumulated degradation products may reinforce the pathological process.

More recently, a growing body of literature indicates that both senescence and the SASP are sensitive to organismal metabolic states, which in turn may drive phenotypes associated with metabolic dysfunction [28,29]. It has been suggested that the metabolic shift of the senescent cells can disrupt the balance between disc ECM anabolism and catabolism; anaerobic metabolism contributes to increased acidity, and, consequently, disc degeneration is accelerated. In fact, the disc microenvironment is complex, as the healthy adult NP is the largest avascular organ in the vertebrate body, and efficient diffusion from penetrating capillaries is the only way to transport nutrients or oxygen and to remove waste products of capillary metabolism [30]. In this context, decreased nutritional intake has been considered a cause of progressive IVDD with aging because of its impact on oxygen and lactic acid transport modes within and outside the disc microenvironment. On the other hand, the transport of nutrients into the disc and, consequently, their concentration in the tissue seem to be positively affected by healthy and active exercise, which may alter the morphology of the capillary bed at the disc–bone interface. As expected, frailty-related physical impairments in the elderly may accelerate IVD degeneration.

From all this evidence, cellular senescence, but especially the different stressors utilizing different signaling networks that determine the senescent phenotype, deserve much attention, as they could be a therapeutic target to combat age-associated intervertebral disc degeneration.

#### 3.1.1. Redox Homeostasis in Intervertebral Discs

##### Mitochondrial Dysfunction

The mitochondrial response to unfolded proteins (UPR^mt^) participates in several aging-related diseases. Recently, Xu et al. [31] explored the role and underlying mechanism of UPR^mt^ in IDD, demonstrating how, in vitro, UPR^mt^ levels and mitophagy were promoted in IL-1β-treated NP cells. They also showed that increased UPR^mt^ inhibited NP cell apoptosis and further enhanced mitophagy. Silencing of PTEN-induced kinase 1 (Pink1), a protein implicated in UPR, reversed the protective effects of nicotinamide riboside (NR) and inhibited UPR^mt^-induced mitophagy. Also, in vivo, NR could attenuate the degree of IDD by activating UPR^mt^ in rats. Additionally, a study on samples from patients undergoing vertebral fusion by Song et al. recently showed how the mitochondrial protein NLR family member X1 (NLRX1) is aberrantly downregulated in degenerated human NP tissues and its expression is closely related to NP cell senescence and exacerbation of disc degeneration. In contrast, mitochondrial collapse occurred in NLRX1-deficient NP cells and activated the Pink1-PRKN (parkin RBR E3 ubiquitin protein ligase) compensatory pathway, leading to excessive mitophagy and aggressive senescence of NP cells [32]. This evidence points in the same direction, suggesting that mitochondrial dysfunction is an excellent target to address IVDD.

##### Glycation End Products in IDD

Since AGEs are known to induce cellular cytotoxicity, they have also been shown to be implicated in the pathogenesis of IDD. Tseng et al. [33] showed how IDD and AGE accumulation were evident in mice on high-AGE diets (HAGE), persisting with dietary changes, but absent in mice on exclusively low-AGE diets. These observations at the cellular and molecular levels were corroborated by the clinical observation of correlation between IDD and diabetes as a risk factor [34].

The effects of AGEs on endoplasmic reticulum (ER) stress, apoptosis, and subcellular calcium (Ca2+) redistribution alter cellular homeostasis and lead to senescence and degenerative damage. Finally, AGEs are thought to be damage mediators fairly high up in the pathophysiological cascade; this is important because antagonizing their production is a key barrier to protecting against IVDD. The argument is even more interesting when considering that good glycemic control can be sufficient to protect against disc damage.

#### 3.1.2. Pyroptosis

Cell death, particularly the death of NP cells, is one of the most critical factors that trigger IVDD. Cell death in this disease context has been shown to occur through a type of programmed cell death that straddles the line between necrosis and apoptosis, called pyroptosis. It is a form of inflammogenic cell death activated by the gasdermine family and often by inflammatory caspases [35,36]. A growing body of evidence has shown that pyroptosis mediated by the NLRP3 inflammasome plays a significant role in IVDD [37,38]. A positive correlation between NLRP3 expression and IVDD was found in 45 clinical samples [39]. The issue is even more complicated, as it has been shown that not only molecules involved in this process called pyroptosis are implicated in cell death, but, depending on the stage of the disease and based on the differential expression profiles of various molecules, other mechanisms of programmed cell death, such as apoptosis, may also be involved [40]. Not only cell death and the resulting inflammatory processes are responsible for IVDD. Since the disc is predominantly made up of ECM, regeneration of this matrix is the greatest protective shield against IVDD. Therefore, it is evident that, in this scenario, the pathological process affects homeostasis that regulates the ECM. For example, the downregulation of nicotinamide phosphoribosyl transferase blocks the priming phase of NLRP3 through the NF-κB and MAPK pathways, reducing aggrecan and collagen II degradation [41]. In general, the DAMPs that are then produced because of the degenerative–inflammatory process reduce ECM synthesis and increase its degradation. In addition to directly altering the balance between ECM synthesis and decomposition, causing NP cell death, inflammatory mediators and cellular contents released by pyroptosis are largely involved in ECM disruption. Indeed, NP cells are crucial in ECM metabolism [42,43].

#### 3.1.3. Autophagy in IDD

Autophagy plays a pivotal role in cellular homeostasis. It is an intracellular degradation process that directs cytoplasmic material to the lysosome for degradation and to derive energy. Nutrient deprivation and hypoxia are the main triggers of autophagy [44]. The role of autophagy in generating or promoting IVDD is only partially understood, and contradictory evidence exists in this regard. Some works have demonstrated that antagonization of autophagy can be an effective tool to prevent and slow down IVDD. For example, in an animal model of IVDD induced by disc damage, modulation of autophagy by metformin led to beneficial effects [44,45]. However, contrary evidence exists as well. Kritschil et al. [46] conducted research on an experimental model of both in vitro and in vivo antagonization of autophagy via bafilomycin A1. They compared the expression profiles of biomarkers of apoptosis and cellular senescence with untreated controls, finding that there was no statistically significant evidence between the treated models and the controls. These controversial results need further experimental investigation and are of extreme interest given the fundamental role that autophagy plays in cellular homeostasis and many models of pathology.

#### 3.1.4. MicroRNAs and IVDD

In recent decades, there has been growing evidence that microRNAs (miRNAs) are deeply involved in the responses of different cells and organs to various mechanical stimuli. Although several miRNAs, such as miR-21 and miR-155, have been reported to influence ECM degradation and promote apoptosis, leading to the development of IVDD, the precise cellular and molecular aspects of damage are unknown [47]. A recent study investigated the role of mechanistically responsive miR-1249, which was identified by miRNA-sequencing as being down-regulated in compression-induced IVDD in rats. It was also revealed that miR-1249 exerted a protective effect on the ECM metabolism of NP. Imaging examination and histological investigation in vivo showed that local injection of vesicles with this miRNA could effectively alleviate the progression of abnormal compressive stress-induced IVDD, including maintenance of water content and disc height, preservation of NP tissues, and amelioration of the imbalance of extracellular matrix metabolism [48]. Also, recently, it was shown that inhibition of miR-96-5p suppressed the progression of IDD by regulating the PPARγ/NF-κB pathway [49]. Another study showed that miR-125b-5p could regulate IDD by regulating NP cell apoptosis and inflammatory responses via TP53-regulated inhibitor of apoptosis 1 (TRIAP1) [50]. Other investigations also showed that miR-423-5p mediates the regulation of NLRX1 to influence apoptosis and ECM levels in IL-1β-induced NP cells [51]. All these miRNAs intersect with the mediators described in the previous sections and, by activating autophagic, mitophagic, and pyrophagic inflammatory damage, could be promising targets for the clinical treatment of IVDD.

#### 3.1.5. Chemokines Rule in IVDD

IVDD is a gradual and persistent pathological process influenced by multiple factors. Chemokines are also involved in this process by regulating the cellular environment and participating in various biological functions, such as matrix synthesis, cell proliferation, apoptosis, and inflammation. They specifically contribute to immune cell recruitment, including macrophages, neutrophils, and microglia, into the degenerated discs. This infiltration and activation of immune cells intensify the inflammatory response and promote the release of neurotrophic factors, further exacerbating the degeneration [52,53,54,55,56].

## 4. Biomarkers

Biomarkers are essential tools for evaluating the diagnosis and monitoring of many clinical conditions. This is especially true even in the context of orphan nosological entities, or other contexts in which the pathophysiological mechanisms are poorly known [57]. The IVDD is often diagnosed from imaging, and its severity is typically objectified from subjective rating scales or other clinical evaluations. The potential role of biomarkers for IVDD can include systemic biomarkers, for example, concerning the blood matrix, and those obtained from histopathology [58].

Concerning systemic biomarkers, there is evidence, for instance, that many cytokines are significantly increased in the blood of subjects with LBP compared with controls. For example, Weber et al. [59] measured the levels of IL-6, IFN-γ, TNF-α, IL-2, IL-3, IL-8, IFN-α2, LIF, MCP-3, and TNF-ß, finding an increase in IDD and spinal stenosis compared with IVDD/herniation. Additionally, Grad et al. [60] measured blood levels of RANTES/CCL5 in a case–control study involving 40 patients and 40 healthy controls, finding that elevated levels correlated with moderate/severe disc degeneration. miRNAs could also be useful biomarkers. In a cohort of 10 patients, Cui et al. [61] found that, compared with the control group, miR-766-3p and miR-6749-3p were upregulated in the blood of IVDD subjects while miR4632-5p was downregulated in the blood of IVDD patients. Further, Divi et al. [62] showed that, in a cohort of 69 patients, miR-155 levels were decreased in patients’ blood serum.

Correlating the radiological finding with the biomarker is a current challenge. In this direction, Aboushaala et al. [63] analyzed 31 subjects, 13 of whom had lumbar Modic changes (MC) and 18 of whom had no MC. Indeed, differences in CCL5 protein and Macrophage Migration Inhibitory Factor (MIF) levels were significantly noted in MC patients compared to those without MC, with *p*-values of 0.028 and 0.030, respectively. Other attempts focused on the combination of MRI image features and inflammatory cascade [58,64,65,66].

## 5. Targeted Pharmacological Therapy

Managing discogenic pain classically involves a multidisciplinary approach with a combination of pharmacological and non-pharmacological therapies [67,68,69]. Given the complex pathophysiology of the disease, targeted pharmacological approaches offer interesting perspectives.

### 5.1. Small Molecule-Based Treatment

Since discogenic pain is strongly related to the patient’s chronic inflammatory response and SASP, the use of small molecules targeting inflammatory mediators involved in ECM degradation is considered a promising therapeutic approach. Most of the evidence comes from in vitro studies reporting that small molecules (natural or chemical/synthetic) can alter a specific signaling pathway or exert their action on multiple targets, sharing common anti-inflammatory and anti-oxidative properties [70].

Notably, some small molecules are able to decrease the levels of inflammatory cytokines and chemokines, including IL-1α, IL-1β, and TNF-α, and increase ECM, also inducing anabolic activity and counteracting catabolism [71].

Natural compounds, including cannabidiol (CBD), naringin, epigallocatechin gallate (EGCG), resveratrol, curcumin, berberine, and icariin, were tested in IVDD cells and demonstrated the ability to reduce levels of IL-1 family and TNF-α, which act as triggers of the NF-kB and p38/MAPK pathways involved in ECM degeneration. Specifically, CBD can inhibit the synthesis of COX2 and IL6, as well as IL-1 family cytokines. Other molecules, either plant supplements, such as luteoloside and icariin, or drugs, such as metformin, appear to be able to suppress IL-1 through inhibition of COX-2 and inducible nitric oxide synthase (iNOS) [72,73,74].

Several studies have also reported the anti-apoptotic effects of small molecules, such as CBD, icariin, and resveratrol, which can increase BcL-2 levels or decrease the expression of caspases 3 and 9, as well as control the release of cytochrome C [75,76]. Resveratrol has also been reported to exert an anti-apoptotic effect in NP cells by activating sirtuin 1 [77]. It is an NAD+ deacetylase known for its anti-inflammatory, anti-oxidative, and anti-senescent effects in the context of disorders such as cardiovascular and respiratory diseases [78,79].

Unlike other molecules that act on multiple targets, some synthetic small molecules appear to inhibit specific signaling pathways: COX2 in the case of celecoxib (both in its original form and as celecoxib-loaded microspheres), NF-kB in the case of gefitinib, and JAK in the case of tofacitinib in vivo [80,81,82]. However, it is important to emphasize that the inhibition of a specific signal pathway may lead to the alteration of downstream biological processes. This is the case with the JAK antagonist tofacinib, which has been reported to significantly reduce COX2 expression and, consequently, PGE2 levels [83].

Although most of the evidence on the effects of small molecules in counteracting IVDD comes from in vitro research, to date, studies have been performed to test the potential benefits of single agents in vivo, including CBD, curcumin, EGCG, statin, gefitinib, and others. Unfortunately, the results are often inconclusive and conflicting due to several reasons, including the inadequacy of the methods used to analyze the regenerative process, the inherent limitations of quadrupedal animal models, the limited follow-up, and the inadequate sample size of the studies [84]. Furthermore, only three retrospective clinical studies have been conducted so far to investigate the effects on LBP of gefitinib [81] and statins [85,86], without reaching any relevant results. To date, these issues complicate the clinical translation of the promising evidence on the use of small molecules in the treatment of IVDD.

### 5.2. Platelet-Rich Plasma-Derived Extracellular Vesicles

Platelet-rich plasma (PRP) is a type of blood-derived product that is produced by centrifugation or the process of apheresis for platelet enrichment of plasma from autologous or allogeneic blood. Its potential has attracted considerable interest in regenerative medicine, especially in the treatment of arthritic conditions of major joints [87]. The function of PRP-derived extracellular vesicles (EV-PRPs), which incorporate a long noncoding RNA (lncRNA), MALAT1, was recently studied as a modifier of IVDD. Indeed, Tao et al. [88] showed that EV-PRPs regulated MALAT1 expression in vivo and in vitro, while downregulation of MALAT1 exacerbated NP cell pyroptosis and ECM degradation. MALAT1 regulated SIRT1 expression through the downregulation of microRNA (miR)-217 in NP cells. SIRT1 blocked pyroptosis mediated by the NF-κB/NLRP3 pathway, thereby alleviating IDD. Evidence in humans is not lacking. The first double-circle randomized clinical trial (RCT) available in the literature included 47 adults with chronic LBP (>6 months), who were randomly assigned to either the treatment group or the control group in a 2:1 ratio [89]. The treatment group received a single injection of L-PRP without activator. The treatment group was given a single injection of L-PRP without activator. At the 8-week follow-up, it was shown that NRS for pain, functional assessment index, and patient satisfaction were significantly improved in the treatment group compared with the control group. In total, 56% (15/27) of participants were satisfied compared with 18% (3/17) of the control participants (3 participants were lost to follow-up). However, outcomes were not compared after 8 weeks because of the lack of follow-up in the control group. No complications were reported. More recently, Li et al. [90] retrospectively analyzed data from 155 patients to evaluate the clinical efficacy and imaging outcomes of percutaneous endoscopic lumbar discectomy (PELD) combined with PRP. The outcomes, such as disc height, were evaluated serially over time and showed statistically significant evidence that PRP injection helped to delay IVDD and promote disc remodeling. Despite the small number of patients and the study design, this treatment is promising for LBP patients with MC type 1 [91].

### 5.3. Stem Cell for IVDD Regeneration

New tissue regeneration therapies offer an interesting perspective [92]. Given the better understanding of the pathophysiology of DLBP, there has been a growing interest in regeneration to restore the initial disc tissue and metabolic balance of the disc matrix through biological means or cell-based therapies [93]. The latter, for example, reimplantation of autologous nucleus pulposus cells, has demonstrated, both in animal models and clinical trials, statistically significant improvements in DLBP scoring, maintenance of IVD hydration, and increased disc height [94]. IVDD causes a switch from type II to type I collagen expression by NP cells and a decrease in aggrecan synthesis. Therapy with multipotent mesenchymal stem cells (MSCs) administered percutaneously has been proposed as a potential means to precisely ameliorate these changes that occur in the degenerated disc. There are essentially three mechanisms by which this occurs: attenuation of primary nociceptive disc pain, slowing or reversal of catabolic metabolism, and restoration of disc tissue. Furthermore, to bridge us to pathophysiology, MSCs have been shown to play an important role in the regulation of pyroptosis and could be useful in alleviating IVDD [95].

Regarding these findings, therapy with allogeneic MSCs can be a viable alternative. A clinical trial demonstrated efficacy in a group of 24 subjects [96]. However, this strategy has limitations, at least in application. A recent study showed that, out of 26 registered studies on the ClinicalTrials.gov repository, only 7 were published. Several factors may contribute to this phenomenon, including inadequate study design, short statistical power, inappropriate eligibility criteria, patient dropout, and financial constraints [97]. Therefore, these translational gaps should be necessarily addressed [98].

## 6. Interventional Techniques

### 6.1. Invasive Percutaneous Procedures

A wide variety of treatments are currently utilized in clinical practice to manage chronic low back pain. However, there remains significant debate and a lack of consensus among clinicians regarding the optimal treatment modality [99]. While interventional pain physicians often employ combinations of structured exercise programs, opioids, epidural injections, intradiscal therapies, and disc surgery, these approaches are frequently accompanied by substantial controversy and differing opinions.

#### 6.1.1. Epidural Injections

Multiple studies, including randomized controlled trials (RCTs), have evaluated the effectiveness of epidural injections in treating discogenic low back pain [100,101]. These studies generally demonstrate a significant association between epidural injections and improvements in both pain and function. The level of improvement is comparable to that seen with epidural injections for disc herniation and is superior to the relief provided for central spinal stenosis [102]. There is an important analysis of efficacy between caudal and lumbar interlaminar injections in discogenic pain. Additionally, epidural injections have been shown to outperform both spinal fusion and disc arthroplasty [103]. The addition of steroids to epidural injections has not been proven to provide superior outcomes compared to steroid-free injections [104]; however, an observational study stipulated that interlaminar epidural injection of steroids performed well for both discogenic pain and patients with low back pain associated with bulging discs [105]. Furthermore, lumbar interlaminar epidural injections have been found to be more effective than caudal epidural injections [106]. Cost utility assessments suggest that the value of epidural injections for discogenic low back pain is comparable to that for disc herniation, central spinal stenosis, and post-surgery syndrome [107]. Overall, the evidence supporting the use of epidural injections in discogenic low back pain is considered moderate.

#### 6.1.2. Percutaneous Intradiscal Therapies

Percutaneous intradiscal therapies are a set of minimally invasive procedures that aim to alleviate discogenic low back pain by reaching inside the intervertebral disc. These therapies use various mechanisms, such as thermal energy, radiofrequency, or chemical agents, to influence disc structure or alter nerve signaling pathways [99].

Therapies using heat are termed as “thermal Annular Procedures”. The aim is to denervate painful nerve fibers and stabilize the disc by causing collagen contraction. Among these, intradiscal electrothermal therapy (IDET) was one of the first to be described; hence, IDET has been extensively studied. The published results remain inconclusive. Indeed, in one RCT, no significant improvement in pain or function was objective compared to sham, and in a second one, pain and function were found to be significantly better; however, the clinical impact was modest [108,109].

Radiofrequency-based therapies can use radiofrequency to coagulate nociceptive nerve fibers within the intervertebral disc. For example, biacuplasty involves the insertion of two probes into the disc to deliver targeted radiofrequency energy. In a placebo-controlled RCT, biacuplasty demonstrated significant improvements in pain, functional status, and disability at six months compared to sham treatment, offering stronger evidence for its use in refractory discogenic pain cases [110].

Another radiofrequency technique, radiofrequency thermocoagulation or “radiofrequency annuloplasty” (often referred to as “disctrode”), uses a single probe producing heat from radiofrequency energy directly to the intervertebral disc. While this method has also been investigated in RCTs, the results have been less conclusive, with some studies showing limited benefits. Systematic reviews such as the one conducted by Helm et al. suggest that, while radiofrequency biacuplasty has strong evidence supporting its efficacy in chronic discogenic pain, the support for radiofrequency thermocoagulation remains limited and less robust [111].

Chemical-based intradiscal therapies include interventions like intradiscal methylene blue injection, which is believed to reduce inflammation and modulate pain pathways [112].

A meta-analysis of five studies found that methylene blue injection significantly reduced pain and improved disability scores in patients with discogenic low back pain. However, more recent evidence from an RCT suggests that methylene blue injections may lack significant clinical efficacy, calling for further research to clarify its role in treatment [112]. Other chemicals are in an early research stage [113].

#### 6.1.3. Spinal Cord Stimulation

Spinal cord stimulation (SCS) is a minimally invasive treatment option for managing chronic refractory pain. In the setting of chronic discogenic low back pain, this treatment has been proven to be effective, safe, and cost-efficient. SCS has been shown to alleviate pain, enhance functional outcomes, and improve patients’ quality of life while also reducing the dependence on pain medications, particularly opioids. A prospective observational study conducted at an urban pain management center confirmed that SCS significantly alleviates pain, decreases disability, and lowers opioid use in patients with discogenic pain [114].

### 6.2. Surgery Lumbar Interbody Fusion

When previous treatments fail to alleviate symptoms of discogenic low back pain, surgical intervention is often considered. Among the surgical options, lumbar interbody fusion (LIF) stands as the most adopted procedure in this scenario [99], as it has been shown to be superior to conservative treatment [115] and it has been investigated through multiple approaches [116,117]. Through a minimally invasive retroperitoneal lateral transpsoas approach, thanks to a series of 28 levels being operated, Marchi et al. [116] found both pain and disability indices to be markedly improved by up to 24 months of follow up, with radiographic evidence of fusion in 93% of cases. Guo et al. [117] reported in a controlled trial that both PLIF with pedicle screw fixation and ALIF with translaminar facet screw have been shown to be more effective than treatment with radiofrequency following successful fusion. However, while lumbar interbody fusion remains the gold standard in these cases, the risk–benefit balance must be carefully weighed due to potential risks such as fusion failure and adjacent segment degeneration, among others.

#### 6.2.1. Prosthesis Disc Replacement

Additional options are available or under development. Prosthesis replacement is one such technique. This relatively new approach involves removing all or part of the disc (specifically the nucleus as mentioned below) and replacing it with a prosthesis. This helps to maintain segmental stability and spinal mobility, restores proper disc height with comparable biomechanical properties, and alleviates inflammation and pain. Notable among these techniques are the prosthetic disc nucleus replacement (PDN) [118] and artificial disc replacement (ADR) [119]. PDN involves only the removal of the nucleus pulposus and its replacement with a prosthesis, requiring a certain integrity of the annulus fibrosus to prevent prosthesis prolapse. ADR involves the total removal of the disc and its replacement, offering a theoretical etiological treatment by removing the inflammatory and autoimmune responses. ADR has been shown to be as effective and safe as fusion in the short to medium term, although long-term data are still lacking [120].

#### 6.2.2. Dynamic Fixation System

Dynamic fixation systems in spinal surgery are classified into non-fusion and fusion dynamic fixation types. Non-fusion dynamic fixation uses implants without bone grafts, allowing for a small range of movement of spinal segments while altering load distribution to control abnormal inter-segmental motion. This method aims to relieve pain and prevent adjacent segment degeneration by maintaining physiological load transfer. Conversely, fusion dynamic fixation stabilizes the lumbar vertebrae and encourages the fusion of the fixed segments, effectively dispersing internal loads. Clinical studies have demonstrated that dynamic fixation systems can significantly improve disability and pain outcomes with an acceptable complication rate, presenting a potential viable option for patients with lumbar spine instability [121].

## 7. Challenges in the Diagnosis and Treatment of Discogenic LBP: A Surgeon’s Perspective

The lack of standardized criteria for the diagnosis of discogenic LBP [122] and the heterogeneity of studies trying to substantiate the evidence for one treatment over another [122] undoubtedly represent stumbling blocks in a spinal surgeon’s decision tree. On the other hand, there are certainly subgroups of patients with LBP who would benefit from surgery. This requires the identification of reliable investigations to identify candidates for surgery. There is some evidence in favor of using lumbar discography to identify pain-generating discs, but this technique remains controversial and is invasive [123]. Even today, a literature search with the two terms “indications” and “techniques”, obviously regarding LBP, returns a dichotomization in which most studies fall into the second category [124], indicating that the most urgent need is to investigate indications in a standardized way, with well-constructed RCTs.

Our opinion is that the limited evidence found in the literature on surgical attitude still entrusts the indication for surgery to the clinical judgment and experience of the individual surgeon. In our personal experience, decompressive spine surgery is widely accepted (albeit with a short evidence base) for the management of conditions associated with neural compression, including refractory radicular leg pain secondary to lumbar disc herniation [125,126]. Somewhat more controversial is the issue of spinal fusion. In this regard, we suggest the introduction of RCTs that consider not only the statistical significance of the data, but also parameters such as the minimum clinically important difference (MCID), a measure that describes the threshold change in an outcome that is clinically, rather than only statistically, significant for patients [127,128]. Still, we must not forget that the correct indications will soon increasingly come through a greater understanding of pathophysiology and through the support of tools such as artificial intelligence that will help us provide increasingly personalized care.

## 8. Future Prospective and AI

Artificial intelligence (AI), emerging from computational sciences and robust databases, uses machine learning (ML) to automatically recognize patterns in data without explicit programming, emulating cognitive functions such as problem-solving and learning. Although AI was conceptualized as early as the 1960s—exemplified by Alan Turing’s famous question, “Can machines think?”—it has witnessed a significant surge in recent years [129]. This surge has been propelled by the advent of advanced models like ChatGPT, made possible by the recent exponential accumulation of big data. AI applications have since expanded across a variety of fields, including finance, autonomous vehicles, and coding. The healthcare sector is no exception, particularly given its vast propensity for data aggregation.

In the context of low back pain (LBP), the earliest mention of AI dates back to 1991, when Mann et al. evaluated the utility of deep learning in pain drawing long before the recent AI boom [130]. Since 2017, however, there has been an explosion of publications in this area, with numerous studies successfully developing AI models that address LBP from diverse perspectives.

### 8.1. AI and Imaging

In the field of medical imaging, AI models have been increasingly focused on the automatic segmentation of MRI sequences. Notably, Huang and colleagues [131] introduced a model for the automatic segmentation of intervertebral discs on MRI, serving as a preliminary step toward further analysis. A systematic review published in 2023 by Compte et al. compiled AI models aimed at aiding in the automatic diagnosis of pathologies related to disc degeneration, herniation, bulging, and Modic changes. This review identified 27 published models, highlighting promising results, but also pointing out methodological limitations likely attributable to the nascent design of these studies [132].

### 8.2. AI for Record Analysis and Interactive Chat

In the area of textual data processing, Ren et al. [133] developed a deep learning algorithm capable of distinguishing between lumbar spine stenosis and lumbar disc herniation by analyzing clinical and radiological records from a cohort of 1921 patients. This was achieved through the application of natural language processing-based machine learning techniques. Similarly, Soin et al. [134] reported on a machine learning model that analyzed pain questionnaires from 246 patients, successfully diagnosing the correct pathology in 72% of low back pain cases.

AI algorithms have also been leveraged for direct patient interaction, providing information through conversational agents such as ChatGPT. Studies evaluating this modality have found that AI-generated responses were generally reliable for answering common, non-patient-specific queries [135]. Additionally, AI has been employed to facilitate surgical triage, predicting a surgeon’s recommendation or the likelihood of surgery based on patient data [136].

### 8.3. AI in Prediction and Surgical Planning

AI has demonstrated utility in preoperative predictions related to postoperative outcomes, such as forecasting serious complications or distant readmissions after lumbar arthrodesis [137]. In terms of surgical planning, AI has been instrumental in determining optimal strategies for addressing degenerative conditions. For example, Purohit et al. [138] employed machine learning models and a prospective cohort to compare treatment approaches for lumbar degeneration, including decompression alone, decompression with fusion, or conservative management with monitoring. Furthermore, Campagner et al. developed an invasiveness score based on inflammatory biomarkers to guide the surgical approach in lumbar fusion procedures for low back pain [139].

## 9. Conclusions

Discogenic LBP is a prevalent and complex condition with significant clinical implications. Advances in understanding the pathophysiology of IDD have provided valuable insights into the multifactorial nature of discogenic pain, highlighting the roles of mitochondrial dysfunction, glycation end products, and pyroptosis. Despite these advancements, diagnostic challenges remain due to overlapping symptoms with other conditions and the lack of definitive criteria.

Current treatment strategies, ranging from conservative approaches to invasive interventions, offer varying degrees of efficacy. Emerging therapies, such as small molecules targeting specific inflammatory pathways, PRP-derived extracellular vesicles, and mesenchymal stem cell-based regeneration, represent promising avenues for future clinical application. However, the translational gap between experimental findings and clinical implementation must be bridged to ensure these innovations reach widespread clinical use.

AI holds potential to revolutionize the diagnosis and treatment of discogenic LBP. Applications of AI in imaging, clinical record analysis, and surgical planning have shown promising results, although further research is needed to optimize these tools for widespread adoption.

In conclusion, a multidisciplinary approach integrating clinical, molecular, and AI-driven data is essential to improve the diagnosis and management of discogenic low back pain, ultimately enhancing patient outcomes.

## References

[1] Low Back Pain Collaborators (2023). Global, regional, and national burden of low back pain, 1990–2020, its attributable risk factors, and projections to 2050: A systematic analysis of the Global Burden of Disease Study 2021. Lancet Rheumatol..

[2] The Lancet Rheumatology (2023). The global epidemic of low back pain. Lancet Rheumatol..

[3] Fatoye F., Gebrye T., Mbada C.E., Useh U. (2023). Clinical and economic burden of low back pain in low- and middle-income countries: A systematic review. BMJ Open.

[4] Fatoye F., Gebrye T., Ryan C.G., Useh U., Mbada C. (2023). Global and regional estimates of clinical and economic burden of low back pain in high-income countries: A systematic review and meta-analysis. Front. Public Health.

[5] Urits I., Burshtein A., Sharma M., Testa L., Gold P.A., Orhurhu V., Viswanath O., Jones M.R., Sidransky M.A., Spektor B. (2019). Low Back Pain, a Comprehensive Review: Pathophysiology, Diagnosis, and Treatment. Curr. Pain Headache Rep..

[6] Knezevic N.N., Candido K.D., Vlaeyen J.W.S., Van Zundert J., Cohen S.P. (2021). Low back pain. Lancet.

[7] Kongsted A., Kent P., Axen I., Downie A.S., Dunn K.M. (2016). What have we learned from ten years of trajectory research in low back pain?. BMC Musculoskelet. Disord..

[8] Chiarotto A., Koes B.W. (2022). Nonspecific Low Back Pain. N. Engl. J. Med..

[9] Remotti E., Nduaguba C., Woolley P.A., Ricciardelli R., Phung A., Kim R., Urits I., Kaye A.D., Hasoon J., Simopoulos T. (2023). Review: Discogenic Back Pain: Update on Treatment. Orthop. Rev..

[10] Malik K.M., Cohen S.P., Walega D.R., Benzon H.T. (2013). Diagnostic criteria and treatment of discogenic pain: A systematic review of recent clinical literature. Spine J..

[11] Lorio M.P., Beall D.P., Calodney A.K., Lewandrowski K.U., Block J.E., Mekhail N. (2023). Defining the Patient with Lumbar Discogenic Pain: Real-World Implications for Diagnosis and Effective Clinical Management. J. Pers. Med..

[12] Raj P.P. (2008). Intervertebral disc: Anatomy-physiology-pathophysiology-treatment. Pain Pract..

[13] Amin R.M., Andrade N.S., Neuman B.J. (2017). Lumbar Disc Herniation. Curr. Rev. Musculoskelet. Med..

[14] Rudnik-Jansen I., van Kruining Kodele S., Creemers L., Joosten B. (2024). Biomolecular therapies for chronic discogenic low back pain: A narrative review. JOR Spine.

[15] Samanta A., Lufkin T., Kraus P. (2023). Intervertebral disc degeneration-Current therapeutic options and challenges. Front. Public Health.

[16] Fearing B.V., Hernandez P.A., Setton L.A., Chahine N.O. (2018). Mechanotransduction and cell biomechanics of the intervertebral disc. JOR Spine.

[17] Fournier D.E., Kiser P.K., Shoemaker J.K., Battié M.C., Séguin C.A. (2020). Vascularization of the human intervertebral disc: A scoping review. JOR Spine.

[18] Marchand F., Ahmed A.M. (1990). Investigation of the laminate structure of lumbar disc anulus fibrosus. Spine.

[19] Johnson W.E., Roberts S. (2003). Human intervertebral disc cell morphology and cytoskeletal composition: A preliminary study of regional variations in health and disease. J. Anat..

[20] Boos N., Weissbach S., Rohrbach H., Weiler C., Spratt K.F., Nerlich A.G. (2002). Classification of age-related changes in lumbar intervertebral discs. Spine.

[21] Daly C., Ghosh P., Jenkin G., Oehme D., Goldschlager T. (2016). A Review of Animal Models of Intervertebral Disc Degeneration: Pathophysiology, Regeneration, and Translation to the Clinic. Biomed Res. Int..

[22] Gao B., Jiang B., Xing W., Xie Z., Luo Z., Zou W. (2022). Discovery and Application of Postnatal Nucleus Pulposus Progenitors Essential for Intervertebral Disc Homeostasis and Degeneration. Adv. Sci..

[23] Buonocore M., Grimaldi M., Santoro A., Covelli V., Marino C., Napolitano E., Novi S., Tecce M.F., Ciaglia E., Montella F. (2023). Exploiting the Features of Short Peptides to Recognize Specific Cell Surface Markers. Int. J. Mol. Sci..

[24] Lopardo V., Montella F., Esposito R.M., Zannella C., Aliberti S.M., Capunzo M., Franci G., Puca A.A., Ciaglia E. (2023). SARS-CoV-2 Lysate Stimulation Impairs the Release of Platelet-like Particles and Megakaryopoiesis in the MEG-01 Cell Line. Int. J. Mol. Sci..

[25] Lyu F.-J., Cui H., Pan H., Mc Cheung K., Cao X., Iatridis J.C., Zheng Z. (2021). Painful intervertebral disc degeneration and inflammation: From laboratory evidence to clinical interventions. Bone Res..

[26] Le Maitre C.L., Freemont A.J., Hoyland J.A. (2007). Accelerated cellular senescence in degenerate intervertebral discs: A possible role in the pathogenesis of intervertebral disc degeneration. Arthritis Res. Ther..

[27] Patil P., Niedernhofer L.J., Robbins P.D., Lee J., Sowa G., Vo N. (2018). Cellular senescence in intervertebral disc aging and degeneration. Curr. Mol. Biol. Rep..

[28] Wiley C.D., Campisi J. (2021). The metabolic roots of senescence: Mechanisms and opportunities for intervention. Nat. Metab..

[29] Ye B., Pei Y., Wang L., Meng D., Zhang Y., Zou S., Li H., Liu J., Xie Z., Tian C. (2024). NAD+ supplementation prevents STING-induced senescence in CD8+ T cells by improving mitochondrial homeostasis. J. Cell. Biochem..

[30] Urban J.P.G., Smith S., Fairbank J.C.T. (2004). Nutrition of the intervertebral disc. Spine.

[31] Xu W.N., Zheng H.L., Yang R.Z., Sun Y.F., Peng B.R., Liu C., Song J., Jiang S.D., Zhu L.X. (2024). The mitochondrial UPR induced by ATF5 attenuates intervertebral disc degeneration via cooperating with mitophagy. Cell Biol. Toxicol..

[32] Song Y., Liang H., Li G., Ma L., Zhu D., Zhang W., Tong B., Li S., Gao Y., Wu X. (2024). The NLRX1-SLC39A7 complex orchestrates mitochondrial dynamics and mitophagy to rejuvenate intervertebral disc by modulating mitochondrial Zn2+ trafficking. Autophagy.

[33] Tseng C., Chen B., Han Y., Wang K., Song Q., Shen H., Chen Z. (2024). Advanced glycation end products promote intervertebral disc degeneration by transactivation of matrix metallopeptidase genes. Osteoarthr. Cartil..

[34] Li S., Du J., Huang Y., Gao S., Zhao Z., Chang Z., Zhang X., He B. (2024). From hyperglycemia to intervertebral disc damage: Exploring diabetic-induced disc degeneration. Front. Immunol..

[35] Luo R., Song Y., Liao Z., Yin H., Zhan S., Wang K., Li S., Li G., Ma L., Lu S. (2019). Impaired calcium homeostasis via advanced glycation end products promotes apoptosis through endoplasmic reticulum stress in human nucleus pulposus cells and exacerbates intervertebral disc degeneration in rats. FEBS J..

[36] Luo J., Yang Y., Wang X., Chang X., Fu S. (2022). Role of Pyroptosis in Intervertebral Disc Degeneration and Its Therapeutic Implications. Biomolecules.

[37] Tang P., Zhu R., Ji W.P., Wang J.Y., Chen S., Fan S.W., Hu Z.J. (2016). The NLRP3/Caspase-1/Interleukin-1β Axis Is Active in Human Lumbar Cartilaginous Endplate Degeneration. Clin. Orthop. Relat. Res..

[38] Zhao K., An R., Xiang Q., Li G., Wang K., Song Y., Liao Z., Li S., Hua W., Feng X. (2021). Acid-sensing ion channels regulate nucleus pulposus cell inflammation and pyroptosis via the NLRP3 inflammasome in intervertebral disc degeneration. Cell Prolif..

[39] Wang J.L. (2015). Enhanced NLRP3, caspase-1, and IL- 1β levels in degenerate human intervertebral disc and their association with the grades of disc degeneration. Anat. Rec..

[40] Li Y., Wu X., Li J., Du L., Wang X., Cao J., Li H., Huo Z., Li G., Pan D. (2022). Circ_0004354 might compete with circ_0040039 to induce NPCs death and inflammatory response by targeting miR-345-3p-FAF1/TP73 axis in intervertebral disc degeneration. Oxidative Med. Cell. Longev..

[41] Huang Y., Peng Y., Sun J., Li S., Hong J., Zhou J., Chen J., Yan J., Huang Z., Wang X. (2020). Nicotinamide Phosphoribosyl Transferase Controls NLRP3 Inflammasome Activity Through MAPK and NF-κB Signaling in Nucleus Pulposus Cells, as Suppressed by Melatonin. Inflammation.

[42] Lawson L.Y., Harfe B.D. (2017). Developmental mechanisms of intervertebral disc and vertebral column formation. Wiley Interdiscip. Rev. Dev. Biol..

[43] Yu X.J., Wang Y.G., Lu R., Guo X.Z., Qu Y.K., Wang S.X., Xu H.R., Kang H., You H.B., Xu Y. (2023). BMP7 ameliorates intervertebral disc degeneration in type 1 diabetic rats by inhibiting pyroptosis of nucleus pulposus cells and NLRP3 inflammasome activity. Mol. Med..

[44] Dikic I., Elazar Z. (2018). Mechanism and medical implications of mammalian autophagy. Nat. Rev. Mol. Cell Biol..

[45] Khaleque M.A., Kim J.-H., Lee H.-H., Kim G.-H., You W.-Y., Lee W.-J., Kim Y.-Y. (2024). Comparative Analysis of Autophagy and Apoptosis in Disc Degeneration: Understanding the Dynamics of Temporary-Compression-Induced Early Autophagy and Sustained-Compression-Triggered Apoptosis. Int. J. Mol. Sci..

[46] Kritschil R., Li V., Wang D., Dong Q., Silwal P., Finkel T., Lee J., Sowa G., Vo N. (2023). Impact of autophagy inhibition on intervertebral disc cells and extracellular matrix. JOR Spine.

[47] Lan T., Shen Z., Yan B., Chen J. (2021). New insights into the interplay between miRNAs and autophagy in the aging of intervertebral discs. Ageing Res. Rev..

[48] Guo J., Yang Y., Ni L., Cao H., Shen H., Luo Z., Niu J., Yang H., Shi Q. (2024). Biomimetic nanovesicles-based therapeutic strategy for alleviating intervertebral disc degeneration via integration with mechanically responsive miR-1249. Nano Today.

[49] Li X., Hou Q., Yuan W., Zhan X., Yuan H. (2023). Inhibition of miR-96-5p alleviates intervertebral disc degeneration by regulating the peroxisome proliferator-activated receptor γ/nuclear factor-kappaB pathway. J. Orthop. Surg. Res..

[50] Jie J., Xu X., Li W., Wang G. (2021). Regulation of Apoptosis and Inflammatory Response in Interleukin-1β-Induced Nucleus Pulposus Cells by miR-125b-5p Via Targeting TRIAP1. Biochem. Genet..

[51] Xu H., Ji L., Yu C., Chen Q., Ge Q., Lu Y. (2020). MiR-423-5p Regulates Cells Apoptosis and Extracellular Matrix Degradation via Nucleotide-Binding, Leucine-Rich Repeat Containing X1 (NLRX1) in Interleukin 1 beta (IL-1β)-Induced Human Nucleus Pulposus Cells. Med. Sci. Monit..

[52] Xue P., Wang Y., Lv L., Wang D., Wang Y. (2024). Roles of Chemokines in Intervertebral Disk De-generation. Curr. Pain Headache Rep..

[53] Li S., Pan X., Wu Y., Tu Y., Hong W., Ren J., Miao J., Wang T., Xia W., Lu J. (2023). IL-37 alleviates intervertebral disc degeneration via the IL-1R8/NF-κB pathway. Osteoarthr. Cartil..

[54] Li H., Pan H., Xiao C., Li H., Long L., Wang X., Luo S., Lyu K., Chen Y., Jiang L. (2023). IL-1β-mediated inflammatory responses in intervertebral disc degeneration: Mecha-nisms, signaling pathways, and therapeutic potential. Heliyon.

[55] Gong Y., Qiu J., Jiang T., Li Z., Zhang W., Zheng X., He Z., Chen W., Wang Z., Feng X. (2023). Maltol ameliorates intervertebral disc degeneration through inhibiting PI3K/AKT/NF-κB pathway and regulating NLRP3 inflammasome-mediated pyroptosis. Inflammopharmacology.

[56] Song X.X., Jin L.Y., Li X.F., Luo Y., Yu B.W. (2021). Substance P Mediates Estrogen Modulation Proin-flammatory Cytokines Release in Intervertebral Disc. Inflammation.

[57] Conti V., Corbi G., Sabbatino F., De Pascale D., Sellitto C., Stefanelli B., Bertini N., De Simone M., Liguori L., Di Paola I. (2023). Long COVID: Clinical Framing, Biomarkers, and Therapeutic Approaches. J. Pers. Med..

[58] Leite Pereira C., Grad S., Gonçalves R.M. (2023). Biomarkers for intervertebral disc and associated back pain: From diagnosis to disease prognosis and personalized treatment. JOR Spine.

[59] Weber K.T., Satoh S., Alipui D.O., Virojanapa J., Levine M., Sison C., Quraishi S., Bloom O., Chahine N.O. (2015). Exploratory study for identifying systemic biomarkers that correlate with pain response in patients with intervertebral disc disorders. Immunol. Res..

[60] Grad S., Bow C., Karppinen J., Luk K., Cheung K., Alini M., Samartzis D. (2016). Systemic blood plasma CCL5 and CXCL6: Potential biomarkers for human lumbar disc degeneration. Eur. Cells Mater..

[61] Cui S., Zhou Z., Liu X., Richards R.G., Alini M., Peng S., Liu S., Zou X., Li Z., Grad S. (2020). Identification and characterization of serum microRNAs as biomarkers for human disc degeneration: An RNA sequencing analysis. Diagnostics.

[62] Divi S.N., Markova D.Z., Fang T., Guzek R., Kurd M.F., Rihn J.A., Hilibrand A.S., Anderson D.G., Vaccaro A.R., Schroeder G.D. (2020). Circulating miR-155-5p as a novel biomarker of lumbar degenerative disc disease. Spine.

[63] Aboushaala K., Chee A.V., Toro S.J., Vucicevic R., Yuh C., Dourdourekas J., Patel I.K., Espinoza-Orias A., Oh C., Al-Harthi L. (2024). Discovery of circulating blood biomarkers in patients with and without Modic changes of the lumbar spine: A preliminary analysis. Eur. Spine J..

[64] Chen X., Wang W., Cui P., Li Y., Lu S. (2024). Evidence of MRI image features and inflammatory biomarkers association with low back pain in patients with lumbar disc herniation. Spine J..

[65] Wang L., He T., Liu J., Tai J., Wang B., Zhang L., Quan Z. (2021). Revealing the Immune Infiltration Landscape and Identifying Diagnostic Biomarkers for Lumbar Disc Herniation. Front. Immunol..

[66] Pelled G., Salas M.M., Han P., Gill H.E., Lautenschlager K.A., Lai T.T., Shawver C.M., Hoch M.B., Goff B.J., Betts A.M. (2021). Intradiscal quantitative chemical exchange saturation transfer MRI signal correlates with discogenic pain in human patients. Sci. Rep..

[67] van Middelkoop M., Rubinstein S.M., Kuijpers T., Verhagen A.P., Ostelo R., Koes B.W., van Tulder M.W. (2011). A systematic review on the effectiveness of physical and rehabilitation interventions for chronic non-specific low back pain. Eur. Spine J..

[68] Costantino M., Izzo V., Conti V., Manzo V., Guida A., Filippelli A. (2019). Sulphate mineral waters: A medical resource in several disorders. J. Tradit. Complement. Med..

[69] Baroncini A., Maffulli N., Schäfer L., Manocchio N., Bossa M., Foti C., Klimuch A., Migliorini F. (2024). Physiotherapeutic and non-conventional approaches in patients with chronic low-back pain: A level I Bayesian network meta-analysis. Sci. Rep..

[70] Vasiliadis E.S., Pneumaticos S.G., Evangelopoulos D.S., Papavassiliou A.G. (2014). Biologic treatment of mild and moderate intervertebral disc degeneration. Mol. Med..

[71] Romaniyanto, Mahyudin F., Sigit Prakoeswa C.R., Notobroto H.B., Tinduh D., Ausrin R., Rantam F.A., Suroto H., Utomo D.N., Rhatomy S. (2022). An update of current therapeutic approach for Intervertebral Disc Degeneration: A review article. Ann. Med. Surg..

[72] Hua W., Zhang Y., Wu X., Kang L., Tu J., Zhao K., Li S., Wang K., Song Y., Luo R. (2018). Icariin Attenuates Interleukin-1beta-Induced Inflammatory Response in Human Nucleus Pulposus Cells. Curr. Pharm. Des..

[73] Lin J., Chen J., Zhang Z., Xu T., Shao Z., Wang X., Ding Y., Tian N., Jin H., Sheng S. (2019). Luteoloside Inhibits IL-1beta-Induced Apoptosis and Catabolism in Nucleus Pulposus Cells and Ameliorates Intervertebral Disk Degeneration. Front. Pharmacol..

[74] Han Y., Yuan F., Deng C., He F., Zhang Y., Shen H., Chen Z., Qian L. (2019). Metformin decreases LPS-induced inflammatory response in rabbit annulus fibrosus stem/progenitor cells by blocking HMGB1 release. Aging.

[75] Deng X., Wu W., Liang H., Huang D., Jing D., Zheng D., Shao Z. (2017). Icariin Prevents IL-1β-Induced Apoptosis in Human Nucleus Pulposus via the PI3K/AKT Pathway. Evid. Based Complement. Alternat. Med..

[76] Chen J., Hou C., Chen X., Wang D., Yang P., He X., Zhou J., Li H. (2016). Protective effect of cannabidiol on hydrogen peroxideinduced apoptosis, inflammation and oxidative stress in nucleus pulposus cells. Mol. Med. Rep..

[77] Wang D., Hu Z., Hao J., He B., Gan Q., Zhong X., Zhang X., Shen J., Fang J., Jiang W. (2013). SIRT1 inhibits apoptosis of degenerative human disc nucleus pulposus cells through activation of Akt pathway. Age.

[78] Conti V., Corbi G., Polito M.V., Ciccarelli M., Manzo V., Torsiello M., De Bellis E., D’Auria F., Vitulano G., Piscione F. (2020). Sirt1 Activity in PBMCs as a Biomarker of Different Heart Failure Phenotypes. Biomolecules.

[79] Conti V., Corbi G., Manzo V., Malangone P., Vitale C., Maglio A., Cotugno R., Capaccio D., Marino L., Selleri C. (2018). SIRT1 Activity in Peripheral Blood Mononuclear Cells Correlates with Altered Lung Function in Patients with Chronic Obstructive Pulmonary Disease. Oxidative Med. Cell. Longev..

[80] Tellegen A.R., Rudnik-Jansen I., Beukers M., Miranda-Bedate A., Bach F.C., de Jong W., Woike N., Mihov G., Thies J., Meij B. (2018). Intradiscal delivery of celecoxib-loaded microspheres restores intervertebral disc integrity in a preclinical canine model. J. Control. Release.

[81] Pan Z., Sun H., Xie B., Xia D., Zhang X., Yu D., Li J., Xu Y., Wang Z., Wu Y. (2018). Therapeutic effects of gefitinib-encapsulated thermosensitive injectable hydrogel in intervertebral disc degeneration. Biomaterials.

[82] Li Z., Gehlen Y., Heizmann F., Grad S., Alini M., Richards R.G., Kubosch D., Südkamp N., Izadpanah K., Kubosch E.J. (2020). Preclinical ex-vivo testing of antiInflammatory drugs in bovine intervertebral degenerative disc model. Front Bioeng. Biotechnol..

[83] Suzuki S., Fujita N., Fujii T., Watanabe K., Yagi M., Tsuji T., Ishii K., Miyamoto T., Horiuchi K., Nakamura M. (2017). Potential Involvement of the IL-6/JAK/STAT3 Pathway in the Pathogenesis of Intervertebral Disc Degeneration. Spine.

[84] Kamali A., Ziadlou R., Lang G., Pfannkuche J., Cui S., Li Z., Richards R.G., Alini M., Grad S. (2021). Small molecule-based treatment approaches for intervertebral disc degeneration: Current options and future directions. Theranostics.

[85] Cheng Y.Y., Kao C.L., Lin S.Y., Chang S.T., Wei T.S., Chang S.N., Lin C.H. (2018). Effect of an increased dosage of statins on spinal degenerative joint disease: A retrospective cohort study. BMJ Open.

[86] Makris U.E., Alvarez C.A., Wei W., Mortensen E.M., Mansi I.A. (2017). Association of Statin Use With Risk of Back Disorder Diagnoses. JAMA Intern. Med..

[87] Xiong Y., Gong C., Peng X., Liu X., Su X., Tao X., Li Y., Wen Y., Li W. (2023). Efficacy and safety of platelet-rich plasma injections for the treatment of osteoarthritis: A systematic review and meta-analysis of randomized controlled trials. Front. Med..

[88] Tao X., Xue F., Xu J., Wang W. (2024). Platelet-rich plasma-derived extracellular vesicles inhibit NF-κB/NLRP3 pathway-mediated pyroptosis in intervertebral disc degeneration via the MALAT1/microRNA-217/SIRT1 axis. Cell Signal..

[89] Tuakli-Wosornu Y.A., Terry A., Boachie-Adjei K., Harrison J.R., Gribbin C.K., LaSalle E.E., Nguyen J.T., Solomon J.L., Lutz G.E. (2016). Lumbar Intradiskal Platelet-Rich Plasma (PRP) Injections: A Prospective, Double-Blind, Randomized Controlled Study. PM&R.

[90] Li T., Du W., Ding Z., Liu J., Ding Y. (2024). Percutaneous endoscopic lumbar discectomy combined with platelet-rich plasma injection for lumbar disc herniation: Analysis of clinical and imaging out-comes. BMC Musculoskelet. Disord..

[91] Kawabata S., Nagai S., Ito K., Takeda H., Ikeda D., Kawano Y., Kaneko S., Shiraishi Y., Sano Y., Ohno Y. (2024). Intradiscal administration of autologous platelet-rich plasma in patients with Modic type 1 associated low back pain: A prospective pilot study. JOR Spine.

[92] Bhujel B., Shin H.E., Choi D.J., Han I. (2022). Mesenchymal Stem Cell-Derived Exosomes and Intervertebral Disc Regeneration: Review. Int. J. Mol. Sci..

[93] Hohaus C., Ganey T.M., Minkus Y., Meisel H.J. (2008). Cell transplantation in lumbar spine disc degeneration disease. Eur. Spine J..

[94] Meisel H.J., Siodla V., Ganey T., Minkus Y., Hutton W.C., Alasevic O.J. (2007). Clinical experience in cell-based therapeutics: Disc chondrocyte transplantation: A treatment for degenerated or damaged intervertebral disc. Biomol. Eng..

[95] Yang S., Zhang Y., Peng Q., Meng B., Wang J., Sun H., Chen L., Dai R., Zhang L. (2024). Regulating pyroptosis by mesenchymal stem cells and extracellular vesicles: A promising strategy to alleviate intervertebral disc degeneration. Biomed. Pharmacother..

[96] Noriega D.C., Ardura F., Hernández-Ramajo R., Martín-Ferrero M.Á., Sánchez-Lite I., Toribio B., Alberca M., García V., Moraleda J.M., Sánchez A. (2017). Intervertebral Disc Repair by Allogeneic Mesenchymal Bone Marrow Cells: A Randomized Controlled Trial. Transplantation.

[97] Ambrosio L., Petrucci G., Russo F., Cicione C., Papalia R., Vadalà G., Denaro V. (2024). Why clinical trials in disc regeneration strive to achieve completion: Insights from publication status and funding sources. JOR Spine.

[98] Seyhan A.A. (2019). Lost in translation: The valley of death across preclinical and clinical divide—Identification of problems and overcoming obstacles. Transl. Med. Commun..

[99] Zhao L., Manchikanti L., Kaye A.D., Abd-Elsayed A. (2019). Treatment of Discogenic Low Back Pain: Current Treatment Strategies and Future Options—A Literature Review. Curr. Pain Headache Rep..

[100] Manchikanti L., Nampiaparampil D.E., Manchikanti K.N., Falco F.J.E., Singh V., Benyamin R.M., Kaye A., Sehgal N., Soin A., Simopoulos T.T. (2015). Comparison of the efficacy of saline, local anesthetics, and steroids in epidural and facet joint injections for the management of spinal pain: A systematic review of randomized controlled trials. Surg. Neurol. Int..

[101] Kaye A.D., Manchikanti L., Abdi S., Atluri S., Bakshi S., Benyamin R., Boswell M.V., Buenaventura R., Candido K.D., Cordner H.J. (2015). Efficacy of epidural injections in managing chronic spinal pain: A best evidence synthesis. Pain Physician.

[102] Manchikanti L., Pampati V., Benyamin R.M., Boswell M.V. (2015). Analysis of efficacy differences between caudal and lumbar interlaminar epidural injections in chronic lumbar axial discogenic pain: Local anesthetic alone vs. local combined with steroids. Int. J. Med. Sci..

[103] Manchikanti L., Staats P.S., Nampiaparampil D.E., Hirsch J.A. (2015). What is the role of epidural injections in the treatment of lumbar discogenic pain: A systematic review of comparative analysis with fusion and disc arthroplasty. Korean J. Pain.

[104] Manchikanti L., Cash K.A., McManus C.D., Pampati V., Benyamin R.M. (2013). A randomized, double-blind, active-controlled trial of fluoroscopic lumbar interlaminar epidural injections in chronic axial or discogenic low back pain: Results of 2-year follow-up. Pain Physician.

[105] Çetin E., Şah V., Zengin I., Arabacı Ö., Akyol M.E., Yücel M. (2023). Comparative Effectiveness of Epidural Steroid İnjections in Patients With Disc Bulging and Disc Protrusion. Cureus.

[106] Manchikanti L., Singh V., Pampati V., Falco F.J.E., Hirsch J.A. (2015). Comparison of the efficacy of caudal, interlaminar, and transforaminal epidural injections in managing lumbar disc herniation: Is one method superior to the other?. Korean J. Pain.

[107] Manchikanti L., Pampati V., Benyamin R.M., Hirsch J.A. (2017). Cost Utility Analysis of Lumbar Interlaminar Epidural Injections in the Treatment of Lumbar Disc Herniation, Central Spinal Stenosis, and Axial or Discogenic Low Back Pain. Pain Physician.

[108] Pauza K.J., Howell S., Dreyfuss P., Peloza J.H., Dawson K., Bogduk N. (2004). A randomized, placebo-controlled trial of intradiscal electrothermal therapy for the treatment of discogenic low back pain. Spine J..

[109] Freeman B.J., Fraser R.D., Cain C.M., Hall D.J., Chapple D.C. (2005). A randomized, double-blind, controlled trial: Intradiscal electrothermal therapy versus placebo for the treatment of chronic discogenic low back pain. Spine (Phila Pa 1976).

[110] Kapural L., Vrooman B., Sarwar S., Krizanac-Bengez L., Rauck R., Gilmore C., North J., Girgis G., Mekhail N. (2013). A randomized, placebo-controlled trial of transdiscal radiofrequency, biacuplasty for treatment of discogenic lower back pain. Pain Med..

[111] Helm Ii S., Simopoulos T.T., Stojanovic M., Abdi S., El Terany M.A. (2017). Effectiveness of Thermal Annular Procedures in Treating Discogenic Low Back Pain. Pain Physician.

[112] Kallewaard J.W., Wintraecken V.M., Geurts J.W., Willems P.C., van Santbrink H., Terwiel C.T.M., van Kleef M., van Kuijk S.M.J. (2019). A multicenter randomized controlled trial on the efficacy of intradiscal methylene blue injection for chronic discogenic low back pain: The IMBI study. Pain.

[113] Rudnik-Jansen I., Tellegen A., Beukers M., Öner F., Woike N., Mihov G., Thies J., Meij B., Tryfonidou M., Creemers L. (2019). Safety of intradiscal delivery of triamcinolone acetonide by a poly(esteramide) microsphere platform in a large animal model of intervertebral disc degeneration. Spine J..

[114] Vallejo R., Zevallos L.M., Lowe J., Benyamin R. (2012). Is spinal cord stimulation an effective treatment option for discogenic pain?. Pain Pract..

[115] Ohtori S., Koshi T., Yamashita M., Yamauchi K., Inoue G., Suzuki M., Orita S., Eguchi Y., Ochiai N., Kishida S. (2011). Surgical versus nonsurgical treatment of selected patients with discogenic low back pain: A small-sized randomized trial. Spine.

[116] Marchi L., Oliveira L., Amaral R., Castro C., Coutinho T., Coutinho E., Pimenta L. (2012). Lateral interbody fusion for treatment of discogenic low back pain: Minimally invasive surgical techniques. Adv. Orthop..

[117] Jun G., Yan W., Zhong-qiang C. (2007). Effect of radiofrequency versus anterior discectomy and posterior lumbar fixation on discogenic low back pain. Zhongguo Zuzhi Gongcheng Yanjiu Yu Linchuang Kangfu.

[118] Wilke H.J., Sciortino V. (2024). The past, present, and the future of disc nucleus replacement. A systematic review of a large diversity of ideas and experiences. Biomaterials.

[119] Bodlák D., Jelen K., Lopot F. (2023). Intervertebral (lumbar) disc replacement: The current state and future perspectives. Neuro Endocrinol. Lett..

[120] Garcia R., Yue J.J., Blumenthal S., Coric D., Patel V.V., Leary S.P., Dinh D.H., Buttermann G.R., Deutsch H., Girardi F. (2015). Lumbar Total Disc Replacement for Discogenic Low Back Pain: Two-year Outcomes of the activL Multicenter Randomized Controlled IDE Clinical Trial. Spine.

[121] Zagra A., Minoia L., Archetti M., Corriero A.S., Ricci K., Teli M., Giudici F. (2012). Prospective study of a new dynamic stabilisation system in the treatment of degenerative discopathy and instability of the lumbar spine. Eur. Spine J..

[122] Ardakani E.M., Leboeuf-Yde C., Walker B.F. (2018). Failure to define low back pain as a disease or an episode renders research on causality unsuitable: Results of a systematic review. Chiropr. Man. Ther..

[123] Manchikanti L., Abdi S., Atluri S., Atluri S., Benyamin R.M., Boswell M.V., Buenaventura R.M., Bryce D.A., Burks P.A., Caraway D.L. (2013). An update of comprehensive evidence-based guidelines for interventional techniques in chronic spinal pain. Part II: Guidance and recommendations. Pain Physician.

[124] Bailey C.S., Rasoulinejad P., Taylor D., Sequeira K., Miller T., Watson J., Rosedale R., Bailey S.I., Gurr K.R., Siddiqi F. (2020). Surgery versus conservative care for persistent sciatica lasting 4 to 12 months. N. Engl. J. Med..

[125] Wilby M.J., Best A., Wood E., Burnside G., Bedson E., Short H., Wheatley D., Hill-McManus D., Sharma M., Clark S. (2021). Surgical microdiscectomy versus transforaminal epidural steroid injection in patients with sciatica secondary to herniated lumbar disc (NERVES): A phase 3, multicentre, open-label, randomised controlled trial and economic evaluation. Lancet Rheumatol..

[126] Harris I.A., Dao A.T. (2009). Trends of spinal fusion surgery in Australia: 1997 to 2006. A. N. Z. J. Surg..

[127] Copay A.G., Glassman S.D., Subach B.R., Berven S., Schuler T.C., Carreon L.Y. (2008). Minimum clinically important difference in lumbar spine surgery patients: A choice of methods using the Oswestry Disability Index, Medical Outcomes Study questionnaire Short Form 36, and pain scales. Spine J.

[128] Evans L., O’Donohoe T., Morokoff A., Drummond K. (2023). The role of spinal surgery in the treatment of low back pain. Med. J. Aust..

[129] Turing A.M. (1950). Computing machinery and intelligence. Mind.

[130] Mann N.H., Brown M.D. (1991). Artificial intelligence in the diagnosis of low back pain. Orthop. Clin. N. Am..

[131] Huang J., Shen H., Wu J., Hu X., Zhu Z., Lv X., Liu Y., Wang Y. (2020). Spine Explorer: A deep learning based fully automated program for efficient and reliable quantifications of the vertebrae and discs on sagittal lumbar spine MR images. Spine J..

[132] Compte R., Granville Smith I., Isaac A., Danckert N., McSweeney T., Liantis P., Williams F.M.K. (2023). Are current machine learning applications comparable to radiologist classification of degenerate and herniated discs and Modic change? A systematic review and meta-analysis. Eur. Spine J..

[133] Ren G., Yu K., Xie Z., Liu L., Wang P., Zhang W., Wang Y., Wu X. (2022). Differentiation of lumbar disc herniation and lumbar spinal stenosis using natural language processing-based machine learning based on positive symptoms. Neurosurg. Focus.

[134] Soin A., Hirschbeck M., Verdon M., Manchikanti L. (2022). A Pilot Study Implementing a Machine Learning Algorithm to Use Artificial Intelligence to Diagnose Spinal Conditions. Pain Physician.

[135] Stroop A., Stroop T., Alsofy S.Z., Nakamura M., Möllmann F., Greiner C., Stroop R. (2023). Large language models: Are artificial intelligence-based chatbots a reliable source of patient information for spinal surgery?. Eur. Spine J..

[136] Broida S.E., Schrum M.L., Yoon E., Sweeney A.P., Dhruv N.N., Gombolay M.C., Yoon S.T. (2022). Improving Surgical Triage in Spine Clinic: Predicting Likelihood of Surgery Using Machine Learning. World Neurosurg..

[137] Berjano P., Langella F., Ventriglia L., Compagnone D., Barletta P., Huber D., Mangili F., Licandro G., Galbusera F., Cina A. (2021). The Influence of Baseline Clinical Status and Surgical Strategy on Early Good to Excellent Result in Spinal Lumbar Arthrodesis: A Machine Learning Approach. J. Pers. Med..

[138] Purohit G., Choudhary M., Sinha V.D. (2022). Use of Artificial Intelligence for the Development of Predictive Model to Help in Decision-Making for Patients with Degenerative Lumbar Spine Disease. Asian J. Neurosurg..

[139] Campagner A., Berjano P., Lamartina C., Langella F., Lombardi G., Cabitza F. (2020). Assessment and prediction of spine surgery invasiveness with machine learning techniques. Comput. Biol. Med..

